# Predictors of e-cigarette initiation and use among middle school youth in a low-income predominantly Hispanic community

**DOI:** 10.3389/fpubh.2022.883362

**Published:** 2022-09-27

**Authors:** M. Yvonne Gaddy, Denise Vasquez, Louis D. Brown

**Affiliations:** Department of Health Promotion and Behavioral Sciences, The University of Texas Health Science Center at Houston, School of Public Health, El Paso, TX, United States

**Keywords:** youth, Hispanic, social cognitive theory, tobacco prevention, e-cigarette initiation, e-cigarette use

## Abstract

**Introduction:**

E-cigarette use among middle and high school youth increased from 2. 5 million in 2014 to 9.2 million in 2019, becoming the most common tobacco product used among youth. Hispanic youth, the largest ethnic minority in the United States, have higher rates of tobacco use, including e-cigarettes, than non-Hispanics. Identifying factors that put youth at risk for future e-cigarette use is vital to focusing prevention efforts. Informed by social cognitive theory, this study identifies predictors of e-cigarette uptake among e-cigarette naïve youth in a predominantly low-income Hispanic community.

**Methods:**

1,249 students (6–8th grades) from two middle schools in El Paso, Texas consented to participate in this longitudinal survey during the 2016–2017 school year. The study sample for analysis was restricted to e-cigarette naïve students (*n* = 862). Outcome measures were e-cigarette initiation and current use at follow-up. Logistic regression models tested six hypotheses about predictors of e-cigarette initiation and current use: (1) intention, (2) outcome expectations, (3) knowledge, (4) friendship network exposure, (5) normative beliefs, and (6) social acceptability.

**Results:**

Among e-cigarette naïve students at baseline, 8% (*n* = 71) reported initiation at follow-up; of these, 3% (*n* = 23) reported current use. Significant predictors of initiation were intention (AOR = 2.46; 95% CI 1.69–3.59; *p* < 0.001), outcome expectations (AOR = 1.73; 95% CI 1.14–2.61; *p* = 0.009), friendship network exposure (AOR = 1.53; 95% CI 1.11–2.11; *p* =0.01), normative beliefs (AOR = 2.12; 95% CI 1.47–3.08; *p* < 0.001), and social acceptability (AOR = 1.91; 95% CI 1.28–2.85; *p* = 0.002). Significant predictors of current use were intention (AOR = 1.98; 95% CI 1.07–3.69; *p* = 0.03) and friendship network exposure (AOR = 1.69; 95% CI 1.06–2.70; *p* = 0.03).

**Conclusions:**

With the increasing popularity of e-cigarettes, age appropriate and culturally sensitive prevention strategies tailored at altering these predictive factors are essential in preventing future e-cigarette use.

## Introduction

Use of electronic cigarettes (e-cigarettes) among middle and high school youth increased from 2.5 million in 2014 to 9.2 million in 2019 (1), becoming the most commonly used tobacco product among youth in the United States (2). Recent findings show that in 2020, 1 in 20 middle school and 1 in 5 high school youth currently use e-cigarettes (3). Much is still unknown about the health effects of e-cigarettes on youth although short-term dangers include wheezing and asthma flares, as well as the potential of e-cigarettes becoming a gateway to other substances (4). Longer-term dangers include addiction, harm to teens' developing brain, and increased risk of developing chronic lung disease (5).

Examining the beliefs that underlie e-cigarette use behavior among adolescents will be beneficial to address this health problem and successfully guide prevention efforts. Thus, this study is informed by social cognitive theory (SCT), which posits that learning occurs in a social context with a three-way reciprocal interaction of the individual, social environment, and behavior and has previously been used to study tobacco use among adolescents (6, 7). Growing literature has documented a myriad of motivating and modifiable factors at varying levels of the socioecological model, particularly the individual and social environmental levels, that put youth at risk for e-cigarette use (8–10).

At the individual level, intention, outcome expectations, and knowledge are factors that affect initiation and sustained use of e-cigarettes (7–13). Previous research found that susceptibility—measured through intention to use a tobacco product in the next year or if offered by a friend—was a strong predictor of uptake of traditional cigarettes and e-cigarettes (7, 9, 11). Outcome expectations—e.g. *If I were to use an e-cigarette, I would enjoy it*—are important determinants of tobacco initiation and subsequent steady use (8, 12). Studies among middle and high school youth found that positive expectations, such as enjoyment, relaxation, or favorable expectancies created by tobacco promotion, were associated with increased initiation of tobacco products (7, 12, 13). High negative outcome expectations, such as health concerns or addiction, influence use although positive expectancies have been found to be more impactful. Knowledge of the potential dangers of e-cigarettes, such as harmful chemicals and addiction, likely deter e-cigarette use (4, 10). On the other hand, beliefs among youth that e-cigarettes are relatively safe and are less harmful than conventional cigarettes has led to increased susceptibility to e-cigarettes (4, 14–16).

At the social environmental level, friendship network exposure, normative beliefs, and social acceptability play important roles in forming youth's decisions regarding risky behaviors, such as e-cigarette use (14, 17). Youth are at a vulnerable age and tend to follow trends set within their social groups. Peer influence has been identified as a leading cause of tobacco use (18, 19). Social environments that accept e-cigarette use foster learning about the various devices and how to use them (16). Normative beliefs that smoking looks cool and increases popularity may motivate initiation (18). In many cases, popular students are likely to have a strong influence in setting these normative beliefs. Social acceptability of what is considered normal, as e-cigarettes currently are, and exposure to e-cigarettes among friendship networks has led to increased e-cigarette initiation and current use among youth (16, 20).

Previous studies have examined the individual and social environmental factors included in our study (intention, outcome expectations, knowledge, friendship network exposure, normative beliefs, social acceptability) and their impact on youth tobacco use (8, 21). But few, if any, have evaluated the combined effects of these factors on both e-cigarette initiation and current use over time among middle school youth, particularly Hispanic youth—the fastest growing minority group in the U.S. (22). Previous research showed evidence of an upward shift in e-cigarette initiation among Hispanic adults from 2017 to 2019 (23), with more recent data indicating similar patterns among Hispanic youth (24). One study using nationally representative data reported increased likelihood of e-cigarette use due to social factors that influence youth (21). More understanding of the combined influence social and individual factors have on both e-cigarette initiation and current use among low-income Hispanic middle school youth would be useful to tailor prevention efforts accordingly.

Hispanic youth report greater intention to use tobacco and e-cigarettes compared to non-Hispanic peers (25) and greater curiosity about e-cigarettes (26). Many Hispanic youth believe that e-cigarette users have more friends, and that e-cigarette use among their friend networks strongly influences their decisions to do the same (21). Initially, e-cigarettes were more widely used among non-Hispanic whites but use among Hispanic youth has steadily increased and in some samples has surpassed usage rates among non-Hispanic groups (27, 28). In recent years, Hispanic middle and high school youth reported higher use of any tobacco product, use of ≥2 tobacco products, and higher use of e-cigarettes compared to non-Hispanic youth, with e-cigarettes the most commonly used tobacco product among Hispanic youth (1, 5, 29). In 2020, 18.9% of Hispanic high school youth and 7.1% of Hispanic middle school youth reported e-cigarette use on at least one or more days in the past 30 days (30). These reports are concerning given that, by comparison, Hispanics are the youngest ethnic group in the U.S. with a large proportion (roughly one third) under the age of 18 (31). Health disparities among Hispanics, in general, are influenced by economic, social, and cultural factors. In the U.S., Hispanics generally have lower levels of education attainment than non-Hispanic whites, are more likely to live in poverty, and are the least likely of any racial or ethnic group to have medical insurance (32). Hispanic adults have experienced greater tobacco-related health disparities—including cancer, heart disease, and stroke—compared to their non-Hispanic counterparts (33, 34). Considering the tobacco-related health disparities and that Hispanics accounted for approximately half (52%) of population growth in the U.S. over the past 10 years (35), it is important that we understand the factors affecting increased e-cigarette use among Hispanic youth.

The purpose of this study is to examine SCT-based predictors of e-cigarette initiation and current use among 6, 7, and 8th grade youth who had never tried e-cigarettes at baseline in a low-income Hispanic community. We hypothesized that significant predictors of e-cigarette initiation and current use at the individual level were (1) intention, (2) outcome expectations, and (3) knowledge; and at the social environmental level, (4) friendship network exposure, (5) normative beliefs, and (6) social acceptability. We examined the hypothesized predictors to identify their unique influence beyond demographic characteristics (e.g. gender, ethnicity, grade, school performance, school) identified as factors related to e-cigarette use among youth (9, 11), as well as the combined influence of significant predictors on initiation and current use.

## Methods

### Study procedures

To identify predictors of e-cigarette initiation and current use over time, we analyzed data collected at baseline and again at follow-up during the 2016–2017 school year as part of an evaluation of Teens Against Tobacco Use (TATU), a school-based peer-led tobacco prevention intervention (36). Two Title I middle schools in El Paso, Texas with students in grades 6–8 enrolled in that study. The schools had a total population of 1,695 students; 95% was Hispanic and 85% received free or reduced-price lunch due to low income. Data were collected during physical education class first in October 2016 (baseline) and again in May 2017 (follow-up) from all participating 6, 7, and 8th graders. Self-report pencil-and-paper surveys, offered in English or Spanish, were administered during physical education class to participating students at both timepoints. Study procedures were approved by the author's Institutional Review Board.

### Participants

Among the 1,695 eligible students, 1,249 (73.7%) returned signed parental consent forms; 1,166 (93.3%) of these students completed surveys at baseline and follow-up. The sample for this analysis, however, was limited to e-cigarette naive students at baseline, defined as never tried an e-cigarette at the time of the baseline survey, with 862 (73.9%) students included in the final sample.

### Measures

The anonymous survey consisted of Likert scale questions drawn primarily from the Pierce et al. measure of smoking susceptibility (9) and the Global Tobacco Youth Survey (37), a self-administered, school-based, public health surveillance system implemented every 4–5 years to monitor the prevalence of tobacco use among middle and high school youth and assess tobacco-related attitudes, knowledge, and behaviors (38). To ensure understandability of the questions and to establish terminology, the survey first provided a simple explanation of electronic cigarettes and commonly used terms—e.g., e-cigarettes, vape, vape pens, etc. —and images of vaping devices, as well as images of regular cigarettes and of hookahs.

#### Outcomes: E-cigarette initiation and current use

E-cigarette initiation was assessed by asking “*Have you ever tried or experimented with electronic cigarettes, even one or two puffs*?” (9). Students who responded “yes” to trying e-cigarettes at follow-up were classified as initiators. Current use was quantified asking “*During the past 30 days, on how many days did you use electronic cigarettes?*”. Those who reported e-cigarette use on at least 1 day over the previous 30 days at follow-up were dichotomized as current user (1 = yes or 0 = no).

#### Independent variables: Individual and social environmental domains

Predictors of e-cigarette initiation and current use were assessed at baseline through a composite of SCT constructs of intention, outcome expectations, and knowledge in the individual domain; and friendship network exposure, normative beliefs, and social acceptability in the social environmental domain.

We assessed each construct on a 5-point Likert scale using multiple questions and calculated the scale mean to create a continuous variable. Intention was assessed through two items (*r* = 0.61), e.g. *Do you think you will use any kind of tobacco product or electronic cigarette in the next year?*; outcome expectations through five items (α = 0.61), e.g. *Do you think you might enjoy smoking an electronic or regular cigarette?*; knowledge through three items (α = 0.56), e.g. *Do you think electronic cigarettes contain harmful chemicals?*; normative beliefs through three items (α = 0.42), e.g. *Do you think smoking looks cool?*; and social acceptability of tobacco use among peers through three items (α = 0.91) asking “*Do you think it is okay for people your age to: Smoke cigarettes? Use e-cigarettes? Smoke hookah?*”. Response options ranged from 1 = “Definitely NOT!”, 2 = “Probably not”, 3 = “Neutral”, 4 = “Probably yes”, and 5 = “Definitely YES!”. Responses for two items under outcome expectations (*I think smoking or vaping would be relaxing* and *I think tobacco would help me deal with problems or stress*) and one item under normative beliefs (*Smoking cigarettes or e-cigarettes is a good way to make friends*.) were on a different 5-point scale, where 1 = “Strongly disagree”, 2 = “Disagree”, 3 = “Neutral”, 4 = “Agree”, and 5 = “Strongly agree”. Friendship network exposure was measured with three items (α = 0.87) asking “*How many of your friends: Smoke cigarettes? Use e-cigarettes? Smoke hookah?*” with response options ranging from 1 = “none” to 5 = “all”.

#### Covariates

Demographic characteristics included in the models were gender, ethnicity, grade (6, 7, 8th), school performance (mostly As, mostly Bs, mostly Cs, mostly Ds, mostly Fs), and school (9). For the analysis, ethnicity was dichotomized into non-Hispanic or Hispanic given that the majority of the students was Hispanic.

### Statistical analysis

Descriptive statistics characterized non-initiators and initiators. Sampling was not classroom dependent and we therefore did not apply multilevel modeling at the classroom level. We instead used logistic regression to test the relation between each hypothesized predictor and e-cigarette initiation at follow-up, controlling for gender, ethnicity, grade, school performance, and school. It should be noted that initial testing for multicollinearity did not produce problematic results. We then fit an additional model with the significant independent predictors (*p* < 0.05) to assess their combined influence on e-cigarette initiation at follow-up. We replicated these models with current use of e-cigarettes at follow-up as the dependent variable, rather than initiation. The amount of missing data was minimal, ranging from 0.006 to 3.5%; therefore, we used listwise deletion, with sample sizes differing minimally across models. We analyzed the data using STATA version 15.1 statistical software.

## Results

Among the 862 e-cigarette naïve respondents at baseline, 8.2% (*n* = 71) reported e-cigarette initiation at follow-up (Table 1); of these, 32.4% (*n* = 23) reported current use. E-cigarette initiation was made up of slightly more females (53.5%; *n* = 38) than males (46.5%; *n* = 33). Grade was the only covariate that significantly differed across participants (*p* < 0.05). Initiation was reported by more 8th grade students (43.7%; *n* = 31) than 7th (31.0%; *n* = 22) and 6th grade students (25.3%; *n* = 18). Most non-initiators (94.7%; *n* = 749) and initiators (90.1%; *n* = 64) reported school performance of mostly A's, mostly B's, and mostly C's. The number of initiators was slightly higher at middle school 2 (57.8%; *n* = 41) versus middle school 1 (42.3%; *n* = 30).

**Table 1:** Key characteristics by e-cigarette status at follow-up (*n* = 862).

**Characteristic**	**Non-initiators** **(*n* = 791)** ***n* (%)**	**Initiators** **(*n* = 71)** ***n* (%)**	***p*-value**
Gender			0.973
Male	366 (46.3)	33 (46.5)	
Female	425 (53.7)	38 (53.5)	
Ethnicity			0.080
Non-Hispanic	89 (11.3)	8 (11.3)	
Hispanic	698 (88.2)	61 (85.9)	
Missing	4 (0.5)	2 (2.8)	
Grade			**0.013**
6th	273 (34.5)	18 (25.4)	
7th	303 (38.3)	22 (31.0)	
8th	215 (27.2)	31 (43.7)	
School performance			0.837
Mostly As	280 (35.4)	24 (33.8)	
Mostly Bs	402 (50.8)	34 (48.0)	
Mostly Cs	67 (8.4)	6 (8.4)	
Mostly Ds	10 (1.3)	2 (2.8)	
Mostly Fs	6 (0.8)	1 (1.4)	
Missing	26 (3.3)	4 (5.6)	
School			0.169
Middle school 1	270 (34.0)	30 (42.3)	
Middle school 2	521 (65.9)	41 (57.8)	

Boldface values indicate statistical significance (p < 0.05). Initiator was defined as “ever tried or experimented with e-cigarettes, even one or two puffs” at follow-up. Of the 71 initiators, 3% (n = 23) reported current use of e-cigarettes (past 30 days), i.e., on at least 1 of the previous 30 days. Key characteristics not shown for e-cigarette current users. Follow-up, May 2017.

### E-cigarette initiation at follow-up

Logistic regression models separately examined the hypothesized predictor of e-cigarette initiation at follow-up (Table 2), controlling for gender, ethnicity, grade, school performance, and school. In the individual domain, two predictors were independently identified as significantly associated with initiation: intention and outcome expectations. The odds of e-cigarette initiation at follow-up were 2.46 times higher for each unit increase in baseline intention scores—e.g., from Probably Yes to Definitely YES! —to use a tobacco or e-cigarette product in the next year (adjusted odds ratio [*AOR*] = 2.46; 95% CI = 1.69–3.59; *p* < 0.001). A one unit increase in baseline scores for outcome expectations—e.g., from Agree to Strongly Agree—predicted 1.73 times higher odds of initiation at follow-up (*AOR* = 1.73; 95% CI = 1.14–2.61; *p* = 0.009). Knowledge was not a significant predictor of initiation (*AOR* = 1.12; 95% CI = 0.83–1.51; *p* = 0.47).

**Table 2:** Regression models predicting e-cigarette initiation at follow-up, with independent variables (IVs) first entered in separate models and significant IVs entered into a combined model (*n* = 862).

**Independent variable (baseline)**	**E-cigarette initiation at follow-up**	
	**Each IV in separate model AOR 95% CI**	**Significant IVs in combined model** **AOR 95% CI**
Individual domain		
Intention	**2.46 1.69–3.59**	**1.86 1.13–3.07**
Outcome expectations	**1.73 1.14–2.61**	0.95 0.55–1.66
Knowledge	1.12 0.83–1.51	–
Social environmental domain		
Friendship network exposure	**1.53 1.11–2.11**	**1.52 1.06–2.19**
Normative beliefs	**2.12 1.47–3.08**	1.50 0.94–2.39
Social acceptability	**1.91 1.28–2.85**	1.20 0.73–1.98

AOR, adjusted odds ratio; all models are adjusted for gender, ethnicity, grade, school performance, and school. IV, independent variable. 95% CI, 95% confidence interval. Bold values indicate statistical significance (p < 0.05). n represents the number of e-cigarette naïve youth at baseline. Baseline, October 2016; Follow-up, May 2017. Only significant IVs in the separate models are included in the combined model; therefore, estimates are not provided for all IVs in the combined model.

In the social environmental domain, normative beliefs, friendship network exposure, and social acceptability were significant predictors of initiation (Table 2). Respondents with higher baseline friendship network exposure to tobacco products had 1.53 times increased odds of initiating e-cigarettes at follow-up (*AOR* = 1.53; 95% CI = 1.11–2.11; *p* = 0.01). Students with higher baseline scores on normative beliefs had 2.12 times higher odds of initiating e-cigarettes at follow-up (*AOR* = 2.12; 95% CI = 1.47–3.08; *p* < 0.001). Those with higher social acceptability of e-cigarette use at baseline had 1.91 times greater odds of initiation at follow-up (*AOR* = 1.91; 95% CI = 1.28–2.85; *p* = 0.002).

When entering only the significant predictors of e-cigarette initiation from the separate models into a combined model (Table 2), intentions and friendship network exposure were significant, whereas outcome expectations, normative beliefs, and social acceptability were non-significant but still associated with increased risk of initiation. A one unit increase in baseline intention scores predicted 1.86 times higher odds of e-cigarette initiation (*AOR* = 1.86; 95% CI = 1.13–3.07; *p* = 0.02). Likewise, a one unit increase in friendship network exposure scores—e.g., from Some to Most—predicted 1.52 times increased odds of initiation (*AOR* = 1.52; 95% CI = 1.06–2.19; *p* = 0.02). McFadden's *R*^2^ ranged from 0.03 to 0.06 in the separate models. When we entered the significant predictors into a combined model, McFadden's *R*^2^ was 0.08.

### E-cigarette current use at follow-up

Following the same analytic process used to predict initiation, we entered the hypothesized predictors into separate logistic regression models to predict e-cigarette current use at follow-up (Table 3), with gender, ethnicity, grade, school performance, and school entered as covariates. Among predictors in the individual domain, intention was significantly associated with current use of e-cigarettes. The odds of current use at follow-up were 1.98 times greater among students with higher baseline intention to use a tobacco or e-cigarette product in the next year or if offered a tobacco product by a best friend (*AOR* = 1.98; 95% CI = 1.07–3.69; *p* = 0.03). Outcome expectations at baseline were not a significant predictor of current use at follow-up (*AOR* = 1.43; 95% CI = 0.69–2.96; *p* = 0.33), nor was knowledge (*AOR* = 0.76; 95% CI = 0.41–1.42; *p* = 0.40).

**Table 3:** Regression models predicting e-cigarette current use at follow-up, with independent variables (IVs) first entered in separate models and significant IVs entered into a combined model (*n* = 862).

**Independent variable (baseline)**	**E-cigarette current use at follow-up**	
	**Each IV in separate model AOR 95% CI**	**Significant IVs in combined model** **AOR 95% CI**
Individual domain		
Intention	**1.98 1.07–3.69**	1.85 1.00–3.44
Outcome expectations	1.43 0.69–2.96	–
Knowledge	0.76 0.41–1.42	–
Social environmental domain		
Friendship network exposure	**1.69 1.06–2.70**	**1.68 1.02–2.77**
Normative beliefs	1.74 0.94–3.23	–
Social acceptability	1.25 0.56–2.79	–

AOR, adjusted odds ratio; all models are adjusted for gender, ethnicity, grade, school performance, and school. IV, independent variable. 95% CI, 95% confidence interval. Bold values indicate statistical significance (p < 0.05). n represents the number of e-cigarette naïve youth at baseline. Baseline, October 2016; Follow-up, May 2017. Only significant IVs in the separate models are included in the combined model; therefore, estimates are not provided for all IVs in the combined model.

In the social environmental domain, friendship network exposure was a significant predictor of e-cigarette current use at follow-up. Students with higher baseline friendship network exposure to tobacco products had 1.69 times increased odds of current use at the end of the school year (*AOR* = 1.69; 95% CI = 1.06–2.70; *p* = 0.03). Normative beliefs at baseline were not a significant predictor of current use at follow-up (*AOR* = 1.74; 95% CI = 0.94–3.23; *p* = 0.08), nor was social acceptability (*AOR* = 1.25; 95% CI = 0.56–2.79; *p* = 0.58).

Upon entering only the significant predictors from the separate models into a combined model, friendship network exposure significantly predicted e-cigarette current use. A one unit increase in baseline friendship network exposure scores predicted 1.68 times higher odds of current use at follow-up (*AOR* = 1.68; 95% CI = 1.02–2.77; *p* = 0.04). Intentions remained associated with a higher risk of current use in the multivariate model (*p* > 0.05). McFadden's *R*^2^ ranged from 0.02 to 0.03 in the separate models and was 0.05 in the combined model.

## Discussion

The current study is of the first to assess combined SCT predictors of both e-cigarette initiation and current use among e-cigarette naïve youth in a low-income predominantly Hispanic community, although more research is needed. The study surveyed two public middle schools representative of the region, which are demographically representative of the neighborhoods served as we had a high response rate within these schools. Key SCT constructs examined at the individual level were intentions, outcome expectations, and knowledge; and at the social environmental level, friendship network exposure, normative beliefs, and social acceptability. The results supported our hypotheses that intentions, outcome expectations, friendship network exposure, normative beliefs, and social acceptability predict significantly higher risk of e-cigarette initiation. In addition, intentions and friendship network exposure at the beginning of the school year predicted significantly higher odds of current use at the end of the school year. The results did not support our hypothesis that knowledge is a significant predictor of e-cigarette initiation or current use; or that outcome expectations, normative beliefs, or social acceptability are significant predictors of current e-cigarette use.

### Individual level

Previous research showed that youth indicating higher intentions to try e-cigarettes predicted the greatest odds of e-cigarette behaviors (39), which is consistent with our findings of intention as a significant predictor of initiation and current use. Curiosity and peer use reportedly increased intentions to try e-cigarettes among e-cigarette naïve middle and high school students (11). Further, intentions were a better predictor of e-cigarette use than race, gender, socioeconomic level, and other tobacco use. Another study found intentions to use e-cigarettes were higher among Hispanic compared to non-Hispanic youth, with curiosity about e-cigarettes an influential factor related to intentions (39). Future research might consider investigating specific factors that influence intentions to use e-cigarettes among Hispanic youth to guide prevention efforts accordingly.

Our findings of outcome expectations as a significant predictor of initiation mirror previous research indicating positive outcome expectations foster attitudes that lead to increased e-cigarette use among youth under age 18 (13, 16). For instance, exposure to tobacco promotion and wanting or owning a promotional item increased susceptibility to tobacco products, including e-cigarettes. Much of the tobacco promotion and advertisements youth see is online and plays a role in making e-cigarettes more attractive. Efforts focused on online and social media messages may be helpful to counter current advertising by providing facts on the dangers of e-cigarettes and of tobacco use in general although more research is needed to create effective messages. Another outcome expectation, enjoyment of e-cigarettes, is a major factor in their appeal to adolescents that possibly overshadows negative consequences (12, 26). One consideration is to reduce the expected enjoyment of e-cigarettes by placing increased emphasis on the harms, such as the consequences of nicotine addiction or the potential for defective e-cigarette batteries to explode (40). Negative outcome expectations, such as health concerns, have been linked to lower odds of e-cigarette initiation and current use (12). However, research also found that negative outcome expectations related to addiction did not influence e-cigarette use among youth, possibly due to disbelief that e-cigarettes are addictive (41). More research is needed to determine influential positive and negative outcome expectations associated with Hispanic youth e-cigarette use.

As reported in other studies among youth (4, 10, 15), our findings did not support our hypothesis that knowledge of e-cigarette dangers significantly influenced initiation or current use. Previous research reported that although adolescents ages 14–18 were aware of e-cigarette risks, knowledge was not a significant factor in their e-cigarette use behavior (10). Many adolescents, regardless of ethnicity, consider e-cigarettes less harmful than traditional cigarettes and cite this as a reason to use these devices (10, 42). Addressing low-risk perceptions may effectively reduce interest. Increased knowledge alone, however, might be insufficient in shifting behavior although it may positively impact other predictors such as outcome expectations. Specifically, increased knowledge of e-cigarette harms—e.g., addiction or chemical dangers—may lead to higher negative outcome expectations and subsequent reductions in initiation and current use. Future research might explore how to make e-cigarette harms more relatable to youth, such as their impact on issues that matter to them like athletic performance or singing.

### Social environmental level

Consistent with previous research suggesting youth's social environment impacts risky behaviors, we found that friendship network exposure to e-cigarettes was a significant predictor of initiation and current use (16, 17, 43), thus addressing social network influences would likely prove beneficial. Programs may optimally be delivered in settings that have social network reach, e.g., schools, given the time youth spend among friends and the influence of school in shaping youth behaviors (44). Other settings to consider are neighborhood community centers or youth clubs. More research on strategies to correct misconceptions about usage rates may be useful, given that beliefs that more friends use e-cigarettes may increase use (45).

Normative beliefs significantly predicted e-cigarette initiation. Perceptions are often influenced by popular others, and beliefs that smoking looks cool and increases popularity have been associated with increased odds of e-cigarette use (17, 21) irrespective of ethnicity, although a stronger association was found among Hispanic youth (21). Youth's social environment may also account for the lack of association between normative beliefs and current use; that is, peer pressure may encourage initiation but not to the point of continued use during middle school. Prevention efforts could address the influence of popularity by creating a climate of undesirability, perhaps through older teens, celebrities, or sports figures. Popular youth might also get involved if offered incentives, such as a certificate, a monetary gift, or extra credit through agreements with the school.

We also found a significant relation between social acceptability and initiation, likely due to the uniqueness of e-cigarette devices or lack of knowledge of the health effects (46). Unfortunately, social acceptance may lead to consistent use of e-cigarettes and potentially of other tobacco products (17). School-based prevention efforts likely provide an opportunity to reach youth's social environment and peer-led programs may deter social acceptability (36). We did not find a significant relation between social acceptability and current use.

When the significant independent predictors of initiation were combined into a single model, intention and friendship network exposure were significantly associated with initiation, and outcome expectations, normative beliefs, and social acceptability became non-significant. Among the two significant predictors of current use—intention and friendship network exposure—only friendship network exposure was significant in the combined model. These results further emphasize the role youth's social environment plays in their decisions to engage in risky behaviors such as e-cigarette use. McFadden's *R*^2^ was higher in the combined models, indicating that these models are better at predicting initiation and current use. Findings also indicate that the predictors under investigation account for overlapping variance in e-cigarette uptake, as the odds ratios of all predictors decreased in the combined model.

### Strengths and limitations

Our findings have important public health implications. The current analyses show the effect individual and social factors have on both e-cigarette initiation and current use among low-income Hispanic youth. Because Hispanics are the largest, fastest growing, and youngest minority group in the U.S. and e-cigarettes are the most used tobacco product among Hispanic youth, study findings may help tailor interventions and messages to reduce e-cigarette uptake among this population.

The methodology enabled us to examine e-cigarette uptake over time. Good response rates suggest the findings are representative of two school catchment areas in El Paso. The theoretically grounded predictors under investigation are not restricted to one level but rather operate at multiple socioecological levels. Our results are comparable to or slightly higher than the nationwide 2016–2017 incidence and prevalence rates among 6–8th grade students. In addition, our numbers are consistent with increasing trends of e-cigarette uptake among Hispanic youth.

Some limitations should also be considered. First, data were self-reported and therefore the findings are subject to response bias, including social desirability. Previous research suggests this bias is relatively limited (47) and that adolescents' self-reporting is a valid and commonly applied method to collect data on risky or sensitive behaviors due to beliefs of greater confidentiality (48). We acknowledge that adolescents' self-reporting is not as accurate as using urinary biomarkers, but past research suggests it corresponds well with biomarkers of tobacco use (49). Second, our sample is unique in that the community is overwhelmingly Hispanic, so trends from areas where Hispanics are the minority may not hold and our results may not generalize to other geographic locations or differing populations. Furthermore, the sample is limited to two schools. Third, e-cigarette uptake among youth may be impacted by factors not included in our study, such as marketing strategies, parental influence, tobacco use by others in the home, community policies, and sociocultural influence. Future research using additional factors might strengthen prevention efforts and may provide valuable insight into patterns of e-cigarette use among youth in general, and among Hispanics in particular. We also did not assess outlet density in our study, which should be a consideration for future research to determine whether there is a relationship between the number of tobacco outlets and e-cigarette uptake among youth (50). A longer study timeframe and tobacco use outcomes might also reinforce the significance of these predictors and may detect additional areas to address. Lastly, Cronbach alpha ranged from 0.42 to 0.91, which was likely due to the number of items, e.g., normative beliefs only has three items, as well as the variance. While the items are positively correlated, further measurement development through the addition of items and additional assessment of the variance may strengthen the reliability of the measures. It should be noted that initial testing for multicollinearity did not produce problematic results.

## Conclusions

Our findings suggest that susceptibility to e-cigarette initiation and current use among predominately Hispanic middle school youth in a low-income community is significantly influenced by factors at the individual and social environmental levels within a socioecological framework. Future research is needed to identify effective ways to educate youth on the harms of e-cigarettes in ways they can relate to and on how e-cigarettes can negatively impact what is important to them. The popularity of e-cigarettes is on the rise among Hispanic youth, the fastest growing minority group in the U.S. For this reason, age appropriate and culturally sensitive prevention strategies tailored at altering these predictive factors may be useful in preventing future e-cigarette initiation and use.

## Data availability statement

The raw data supporting the conclusions of this article will be made available by the authors, without undue reservation.

## Ethics statement

The studies involving human participants were reviewed and approved by Institutional Review Board of the University of Texas Health Science Center at Houston. Written informed consent to participate in this study was provided by the participants' legal guardian/next of kin.

## Author contributions

LB: conceptualized the study. DV: led the investigation process. MG: analyzed data, led the paper writing and revising with the contributions from LB and DV. All authors read and approved the final draft.

## Funding

Data collection and preliminary analysis were supported by the Paso del Norte Health Foundation through A Smoke-Free Paso del Norte initiative. Additionally, preparation of this article was supported, in part, by the National Cancer Institute through a Community Networks Program Center Grant (U54 CA153505).

## Conflict of interest

The authors declare that the research was conducted in the absence of any commercial or financial relationships that could be construed as a potential conflict of interest.

## Publisher's note

All claims expressed in this article are solely those of the authors and do not necessarily represent those of their affiliated organizations, or those of the publisher, the editors and the reviewers. Any product that may be evaluated in this article, or claim that may be made by its manufacturer, is not guaranteed or endorsed by the publisher.

## Abbreviations

SCT: social cognitive theory
AOR: adjusted odds ratio
CI: confidence interval
IV: independent variable.
